# Optical Coherence Tomography Angiography Findings in Long-Term Follow-up of Leber’s Hereditary Optic Neuropathy: Report of Two Cases

**DOI:** 10.4274/tjo.galenos.2020.86300

**Published:** 2020-10-30

**Authors:** Pınar Bingöl Kızıltunç, Feyza Tüntaş Bilen, Huban Atilla

**Affiliations:** 1 Ankara University Faculty of Medicine, Department of Ophthalmology, Ankara, Turkey; 2 Haymana State Hospital, Clinic of Ophthalmology, Ankara, Turkey

**Keywords:** Leber’s hereditary optic neuropathy, optical coherence tomography, optical coherence tomography angiography, retinal nerve fiber layer

## Abstract

Leber’s hereditary optic neuropathy (LHON) is thought to be a neurovascular disease due to presence of vascular changes in asymptomatic patients. Here we present 2 patients in whom optical coherence tomography angiography (OCTA) imaging demonstrated capillary drop-out areas and decreased radial peripapillary capillary (RPC) density in the quadrants that had thinner retinal nerve fiber layer (RNFL) in OCT images. Progressive decrease in RNFL and RPC density were shown in each patient at month 12 and 30 of follow-up. Following up patients with OCTA imaging in the future will provide insight into the pathogenesis and prognosis of LHON.

## Introduction

Leber’s hereditary optic neuropathy (LHON) is a maternally inherited mitochondrial DNA-related disease.^1^ Optic disc hyperemia, vascular tortuosity, and peripapillary telangiectatic vessels are characteristic findings in asymptomatic patients and patients with early-stage disease. Increased vessel tortuosity and capillary size at the optic disc suggest that the disease is a neurovascular disorder.^2^ Recently, the development of optical coherence tomography angiography (OCTA) has enabled noninvasive evaluation of the ocular microvasculature. With this method, the peripapillary retinal and vascular circulation can be evaluated three-dimensionally. Evaluation of vascular changes in LHON using OCTA may help us to understand the pathophysiology of the disease, assess disease progression, and monitor the efficacy of treatment. Here we presented the optical coherence tomography (OCT) and OCTA findings of 2 patients with LHON.

## Case Report

### Case 1

**Figure 1:**
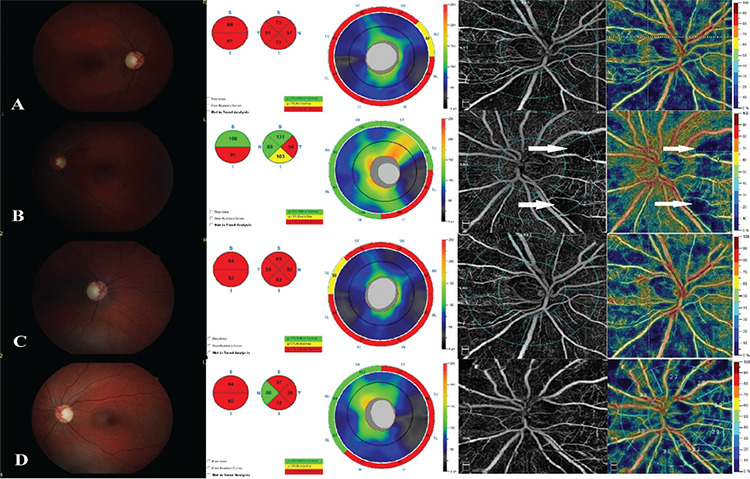
Fundus photos, optic disc OCT and OCTA findings of patient 1. A, B) Findings of the right (A) and left (B) eye at initial examination. From left to right: Optic disc pallor is seen in fundus photograph; decrease in thickness of RNFL on OCT; peripapillary capillary drop-out areas (arrows) and decrease in RPC density on OCTA. C, D) Findings of right (C) and left (D) eye at 12-month follow-up examination. From left to right: optic disc pallor is seen in fundus photograph; marked decrease in thickness of RNFL on OCT; increased peripapillary capillary drop-out areas and marked decrease in RPC density on OCTA RNFL: Retinal nerve fiber layer, OCT: Optical coherence tomography, OCTA: Optical coherence tomography angiography, RPC: Radial peripapillary capillary

A 28-year-old man with no relevant history was referred to our clinic with loss of vision in both eyes, starting first in his right eye and 1 week later in the left eye. His best corrected visual acuity (BCVA) was counting fingers at 25 cm in the right eye and 1/20 in the left eye. Optic discs were pale in both eyes. OCT showed thinner retinal nerve fiber layer (RNFL) in all quadrants in the right eye and consistent with the OCT findings, there were areas of capillary drop-out and decreased radial peripapillary capillary (RPC) density in all quadrants on OCTA (AngioVue RTVue XR Avanti with AngioVue, OptoVue, Inc, Fremont, CA). The RNFL was thinner in the inferior and temporal quadrants and, similar to OCT findings, capillary drop-out areas and decreased RPC density were detected in the inferior and temporal quadrants in the left eye (Figure 1). Genetic testing confirmed the diagnosis with G11778A mutation. Idebenone treatment (900 mg/day) was given orally. Progressive decreases in RNFL and RPC density were shown at month 12 of follow-up (Figure 1).

### Case 2

**Figure 2:**
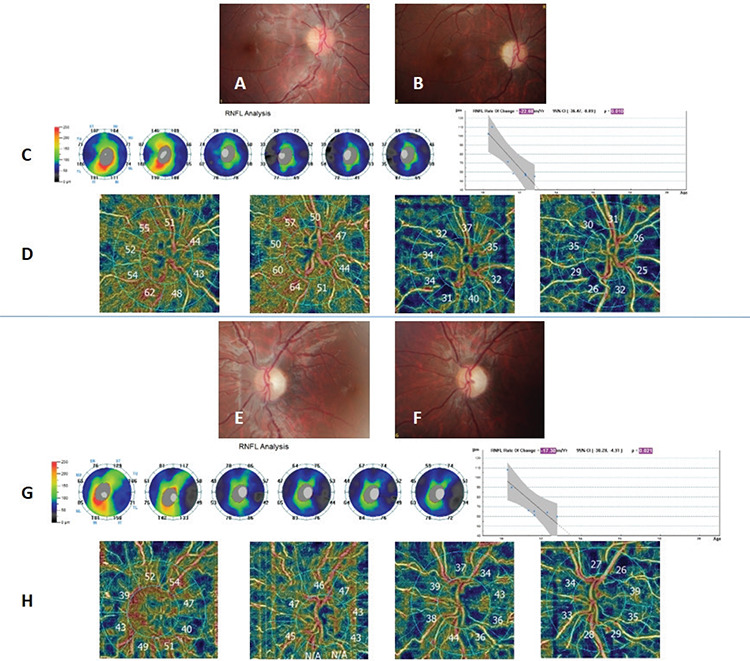
Progressive changes in the optic disc during a 30-month follow-up period in both eyes of patient 2. A) Normal optic disc findings of the right eye in the first examination; B) Optic disc pallor in the right eye in the last examination; C) Progressive decrease in the RNFL in the right eye on OCT; D) Progressive decrease in RPC density in the right eye on OCTA. E,F) Optic disc pallor in the first and last examination in the left eye; G) Progressive decrease in RNFL in the left eye on OCT; H) Progressive decrease in RPC density in the left eye on OCTA RNFL: Retinal nerve fiber layer, OCT: Optical coherence tomography, OCTA: Optical coherence tomography angiography, RPC: Radial peripapillary capillary

An 8-year-old boy with family history of LHON in his maternal uncle was examined. Ophthalmologic examination revealed normal findings. Although the family was informed, they did not consent to genetic evaluation. Two years later, the patient presented with loss of vision in his left eye. BCVA was 20/20 in the right eye and counting fingers at 1 meter in the left eye. Optic discs were normal in the right eye and pale temporally in the left eye. He was diagnosed as having LHON with G11778A mutation and idebenone (450 mg/day) treatment was given orally. At 6-month follow-up, the right eye was also affected. Visual acuities were 2/20 in the right eye and counting fingers at 1 m in the left eye, and both optic discs were pale temporally. The patient was followed up with idebenone therapy OCT and OCTA imaging for 30 months. Visual acuity was counting fingers at 1 m and there was optic disc pallor in the both eyes at the last visit. Although his right eye was not affected in the first visit, drop-out areas were determined by OCTA imaging. Decreased RNFL thickness and RPC density and increased capillary drop-out were shown by OCT and OCTA in the follow-up period (Figure 2).

## Discussion

Since OCTA imaging is a noninvasive and easy method to evaluate vascular structures, different studies evaluated vascular changes in LHON patients and demonstrated RPC defects.^3,4^ Balducci et al.^4^ reported decreases in RPC mostly in the temporal region in patients at different stages.

Our case reports have importance for evaluating RPC density and capillary drop-out areas in LHON over long-term follow-up and we were also able to compare OCTA findings in patient 2 before and after involvement. In both cases, in parallel to the decrease in RNFL, RPC density decreased and peripapillary capillary drop-out areas increased.

In our first case, both eyes were affected when the patient presented to our clinic. A decrease in RNFL was observed in all quadrants in the right eye, which was involved first, while the left eye was affected later and the decrease in RNFL was shown only in the temporal and inferior quadrants. Decrease in RPC density was correlated with loss of RNFL. RPC density was decreased in all quadrants in the right eye and in the temporal and inferior quadrant in the left eye in the first evaluation. In the early stage of LHON, it is known that the smaller caliber fibers of papillomacular bundle are lost, and then loss of fibers progresses and all fibers of the optic nerve can be affected.^5,6^ In accordance with this knowledge, loss of RNFL and RPC density were detected in the inferior and temporal quadrant in the left eye in the early stage of disease. In the follow-up period, both RNFL and RPC density decreased progressively in all quadrants.

In the second case, we were able to evaluate the OCT and OCTA findings of the right eye before involvement and we detected drop-out areas. Before visual acuity decreased in the right eye, we showed that there were drop-out areas. This finding is important in terms of demonstrating OCTA changes in asymptomatic carriers. In the acute phase of the right eye, RNFL thickness increased and visual acuity decreased without any change in RPC density. Decrease in RPC density followed the decrease of RNFL. In LHON, it has been shown that RNFL thickness increases in the early acute phase (within 12 weeks of symptom onset) and decreases gradually in the late acute (12 to 24 weeks after symptom onset) and chronic (24 weeks or more after symptom onset) phases.^7^ The increase in thickness can be explained by impaired axoplasmic transport and a compensatory increase of mitochondrial biogenesis. It is followed by progressive thinning as atrophy develops in the later stages. Decrease in RPC density can be associated with the thinning of RNFL. When the RNFL get thinner, the required metabolic activity is reduced and this can explain the progressive decrease of RPC density in LHON as the disease progresses and RNFL get thinner.

In the follow-up period of our patients, although the decrease in RPC density progressed, visual acuity was stable. This may be explained by macular microvascular changes. In LHON patients, not only the optic disc but also macular microvascular structures are affected. Borrelli et al.^8^ determined that the severity of visual loss was associated with the density of macular superficial capillary plexus. A shortcoming of these case reports is that macular microvascular changes were not evaluated.

In conclusion, OCTA imaging is thought to be an important test for evaluating changes in LHON patients and asymptomatic carriers. In the future, following patients with treatment will help us to gain insight into the pathogenesis and prognosis of LHON.
